# B7-H3 is widely expressed in soft tissue sarcomas

**DOI:** 10.1186/s12885-024-13061-4

**Published:** 2024-10-30

**Authors:** Meghan M. Lynch, Rusul Al-Marayaty, Farres Obeidin, Borislav A. Alexiev, Eleanor Y. Chen, Pedro Viveiros, Brett A. Schroeder, Kelly Hudkins, Timothy M. Fan, Mary W. Redman, Kelsey K. Baker, George Jour, Lee D. Cranmer, Seth M. Pollack

**Affiliations:** 1 Department of Internal Medicine, Northwestern University, Chicago, IL USA; 2 Department of Medicine, Division of Oncology, Northwestern University, 303 E. Superior St. #3-115, Chicago, IL 60611 USA; 3 Department of Pathology, Northwestern University, Chicago, IL USA; 4 Department of Laboratory Medicine and Pathology, University of Washington, Seattle, WA USA; 5 National Cancer Institute, NIH, Bethesda, MD USA; 6 Department of Veterinary Clinical Medicine, University of Illinois at Urbana-Champaign, Champaign, IL USA; 7 Department of Clinical Biostatistics, Fred Hutchinson Cancer Center, Seattle, WA USA; 8 Department of Pathology, New York University, New York, NY USA; 9 Cancer Center at Illinois, University of Illinois at Urbana-Champaign, Champaign, IL USA; 10 Division of Medical Oncology, University of Washington and Clinical Research Division of the Fred Hutchinson Cancer Center, Seattle, WA USA

**Keywords:** B7-H3, Sarcoma, Soft tissue sarcoma, Immunotherapy, Therapeutic target

## Abstract

**Purpose:**

Targeted therapy development in soft tissue sarcoma (STS) has been burdened by the heterogeneity of this group of rare tumors. B7 homolog 3 protein (B7-H3) is a molecule in the same family as programmed death-ligand 1 (PD-L1). It has limited expression in noncancerous tissues and is overexpressed in many cancers, making it an attractive target for cancer therapy, and clinical trials targeting B7-H3 are actively underway. While available data demonstrate high expression levels of B7-H3 in individual sarcoma subtypes, its expression patterns across STS subtypes are not well described. The purpose of this study was to characterize the expression patterns of B7-H3 in STS.

**Patients and methods:**

This retrospective analysis evaluated STS tumor specimens from patients with a variety of different subtypes. Specimens were evaluated by immunohistochemistry (IHC) for expression and staining pattern of B7-H3 both in tumors and in associated vasculature.

**Results:**

Specimens from 153 sarcoma patients included 15 different STS subtypes. B7-H3 was broadly expressed in 97% of samples (95% CI 0.93–0.99) and 69.2% demonstrated high levels of B7-H3 expression (95% CI 0.61–0.76). No significant association between B7-H3 positivity or expression level and prior treatment(s), tumor size, tumor grade, or patient age. B7-H3 positivity in vessels was found in 94.7% (145/153) of samples. In tumors that had been previously assessed for PD-L1 and PD-1, there was no correlation between B7-H3 positivity or expression and the positivity or expression level of PD-L1 or PD-1.

**Conclusion:**

These data show high levels of B7-H3 positivity across soft tissue sarcoma subtypes, suggesting its feasibility as a therapeutic target for future sarcoma treatments. Future clinical trials are needed to evaluate whether targeting B7-H3 can provide clinical benefit to help patients with sarcoma.

**Supplementary Information:**

The online version contains supplementary material available at 10.1186/s12885-024-13061-4.

## Introduction

Sarcomas are a rare group of tumors encompassing > 50 distinct histologic subtypes [1]. These tumors can originate from a variety of tissues, such as fibrous tissue, adipose tissue, smooth or striated muscle, cartilage, or bone. Each subtype presents a unique clinical behavior and biology, making it difficult to institute treatment strategies effective toward sarcoma malignancies as a group. In addition, sarcomas only encompass approximately 1% of adult malignancies, making their rarity a hurdle in studying these tumors [2]. 

Sarcomas arising from bone are classified separately from soft tissue sarcomas (STS) due to their differences in clinical presentation, patient population commonly affected, and clinical response to therapies. Due to the infiltrative nature of STS, surgical resection for localized tumors is often challenging. Even when primary STS tumors are successfully treated, 25–50% of patients will experience relapse with distant metastatic disease [2]. In the metastatic setting, overall survival remains at approximately 16–24 months despite extensive efforts within the field to improve treatment efficacy [3]. 

There is a tremendous need for novel, effective therapies for STS patients in the advanced setting [4]. The most common non-gastrointestinal stromal tumor (GIST) STS subtypes are liposarcoma (LPS), leiomyosarcoma (LMS), and undifferentiated pleomorphic sarcoma (UPS), which together comprise approximately half of all non-GIST sarcoma cases. Other sarcoma subtypes include synovial sarcoma (SS), angiosarcoma (AS), alveolar soft part sarcoma (ASPS), and myxofibrosarcoma (MFS), with the many remaining ultra rare subtypes comprising around one-third of all STS malignancies [5]. 

The success of cancer immunotherapy in other types of cancer, such as melanoma, lung cancer, and renal cancers, has driven investigation into the role of immunotherapy and its potential applications in sarcoma. Immune checkpoint inhibitors such as atezolizumab, pembrolizumab, and nivolumab have shown occasional responses when used as monotherapy in certain sarcoma subtypes, such as UPS, ASPS, and LPS, and improved success when used in combination with other therapies in additional subtypes, such as AS [4, 6–10]. 

B7 homolog 3 (B7-H3) is a molecule in the same family as PD-L1 (also known as B7H1). Since it was discovered, the role of B7-H3 in tumor cells has been investigated by many, and its function in tumors remains unknown [11], though some have suggested that it may play an immunosuppressive role within the tumors as its expression has been associated with decreased T cell infiltration as well as T cell exhaustion [12–15]. Despite research efforts, the cognate receptor for B7-H3 also remains unknown and B7-H3 is often referred to as an “orphan ligand.” [12–14].

Despite the lack of clarity regarding its function, B7-H3 clearly has strict post-transcriptional regulation limiting its expression in non-tumor tissues. It is overexpressed in numerous tumor types, making it an attractive cell surface target for treatments such as antibody based therapies including monoclonal antibodies without additional modification, antibody-drug conjugates, bi-specifics and radio-conjugated antibodies, as well as cell therapies using chimeric antigen receptors [16–19].

B7-H3 expression has been studied in various cancers, including breast cancer, lung cancer, gastric cancer, and squamous cell carcinoma; its presence has been correlated with inferior survival and increased recurrence rates [20–22]. Using immunohistochemistry (IHC), prevalent B7-H3 expression has been recognized in multiple sarcoma subtypes, including rhabdomyosarcoma, osteosarcoma, liposarcoma, Ewing’s sarcoma, synovial sarcoma, and chondrosarcoma [23–25]. This study was conducted to further explore B7-H3’s expression patterns across sarcoma subtypes and the relationship, if any, with PD-1 and PD-L1 expression. To our knowledge, this is the largest investigation of B7-H3 expression in sarcomas to date.

## Materials and methods

### Study design and participants

This study was approved by the Fred Hutchison Cancer Center Institutional Review Board. It is a retrospective study using de-identified archived tissue samples from adult sarcoma patients treated at the University of Washington (Seattle, WA) and performed in concordance with the principles outlined in the Declaration of Helsinki. All tumors were either included previously as part of one of two previously published prior studies, which analyzed the sarcoma tumor immune environment but did not look specifically at B7-H3 expression [26, 27], or they were looked at as part of an internal effort to analyze the tumor microenvironment assisting with clinical trial design [15, 28, 29]. Samples were included regardless of clinical setting including prior treatment, whether the patient had localized or metastatic disease, intent whether it was curative or palliative and regardless of the specimen’s origin whether it was a biopsy or a resection. There was no need for a waiver of consent since all subjects and samples were de-identified. Furthermore, Human Ethics and Consent to Participate declarations are not applicable in our study. Specimens were previously obtained during routine course of clinical treatment by surgical biopsy or at autopsy from patients with established diagnoses by an experienced bone and soft tissue pathologist.

## Procedures

Detailed methods for B7-H3 immunohistochemistry (IHC) staining were published previously [16]. Briefly, automated staining was performed on 4-mm-thick formalin-fixed paraffin embedded (FFPE) sections. Slides were incubated for 1 h with primary antibodies against B7-H3 (clone RBT-B7-H3) which has been used and validated in prior studies [30, 31], followed by the secondary antibody (PerkinElmer OPAL Polymer HRP Ms Plus Rb) for 30 min. Staining was performed with 3,30-diaminobenzidine or fluorochrome. The tertiary TSA-amplification reagent PerkinElmer OPAL fluor was then applied for 10 min followed by antigen stripping. Slides were then imaged with a Leica SP8 confocal microscope or scanned by an Aperio Scanscope. Slides were evaluated for B7-H3 expression, vessel positivity for B7-H3, and B7-H3 staining pattern. PD-L1 and PD-1 expression were not done for this study specifically, but prior results were combined and analyzed together with the B7-H3 expression data. All PD-L1 and PD-1 scoreing was done previously, no new staining or scoring was done for this study; the staining and scoring procedures used for all specimens included here were previously described [26]. 

As a variety of methods are used for scoring B7-H3 IHC staining [32, 33]; with methods ranging from binary “positive vs. negative” to semi-quantitative percentage scoring as we have done in this study. B7-H3 Expression was initially scored from 0 to 5, with a score of 0 representing negative staining and scores 1–5 representing increasing levels of tumor expression with respect to frequency. Vessel positivity was defined as any identification of B7-H3 in the vessels, regardless of the level of expression. Furthermore, B7-H3 pattern in tumor cells was assessed based on the presence of membranous or membranous and cytoplasmic positivity. which is the typical expected staining pattern B7-H3. Faint cytoplasmic or nuclear blush staining alone was not counted. The percentage given refers to overall amount of tumor staining on one representative slide of tumor stained with B7-H3. All B7-H3 staining was reviewed by two pathologists (GJ and FO) and results were only included when they were in concordance.

### Statistical analysis

Patient demographics and tumor characteristics were summarized for all patients using frequencies and percentages or means and ranges for categorical and continuous variables, respectively. For analysis purposes, B7-H3, PD-1, and PDL-1 expression were each broken down into two binary groups based on expression score: negative vs. positive and low vs. high. For all three molecules, a score equal to zero was considered negative expression, whereas a score greater than zero was considered positive expression. For B7-H3, a score less than three (< 3) was considered low expression (< 50% of the tumor stains with B7-H3), and a score greater than or equal to three (≥ 3) was considered high expression (> 50% of the tumor cells positively stain with B7-H3). For both PD-1 and PDL-1, a score less than two (< 2) was considered low expression, while a score greater than or equal to two (≥ 2) was considered high expression. The association of B7-H3 with patient demographics and tumor factors was evaluated using the Fisher’s Exact Test for categorical variables and the linear model ANOVA for continuous variables. The correlation between B7-H3, PD-L1 and PD-1 was calculated using the Fisher’s Exact Test. Survival estimates were calculated using Kaplan-Meier method. Survival time was calculated from the date of diagnosis to the date of death. Patients last known to be alive were censored at the date of last contact. No adjustments were made for multiple comparisons. All reported p-values are two-sided, and statistical significance was based on a two-sided alpha level of 0.05 wherein a P value < or equal to 0.05 was considered significant. Statistical analyses were performed using SAS Statistical Software Version 9.4.

## Results

### Patient characteristics

Tissue samples from 156 patients were included in the study. Tumor samples were all reviewed by two pathologists (JG and FO) and tumors were only included if there was full concordance in their assessment. Concordance was seen in 153 samples (99%) and only these samples were included for further analysis.

Patients ranged from 14 to 86 years old (average 47 years) at the time of diagnosis, though all were adults (age ≥ 18) when they were seen at the University of Washington and all samples used were taken when patients were adults. Histologic STS subtypes are summarized in Table 1. Forty-nine patients (32.03%) had LPS, 32 (20.92%) had SS, 34 (22.22%) had LMS, 25 (16.34%) had UPS, and 13 (8.5%) had less common sarcoma subtypes (Table 1, Appendix1). Approximately half (47.06%) of the patients were untreated at the time of tissue sampling, while 25.49% had received chemotherapy only, 4.58% had received radiation only, and 21.57% had received chemotherapy plus radiation (Table 2). The average tumor size was 9.71 cm (range 1.2–40 cm), and the majority of patients had grade 2 or 3 tumors (35.95% and 39.22%, respectively). Almost 30% of the patients had local recurrence, and 39.87% developed metastatic disease. At the time of data collection, 52.3% of patients were still alive. Baseline demographics are summarized in Tables 1 and 2.

**Table 1:** Patient demographics focusing on histology

Variable	Value	%
**Age at Diagnosis (Years)**		
Mean (Range/SD)	47.9 (14.1–86.3/16)	
Missing Data	37	
**Tumor type**	*n* = 153	
Leiomyosarcoma (LMS)	34	22.2%
Liposarcoma (LPS)	49	32.0%
Undifferentiated Pleomorphic Sarcoma (UPS)	25	16.3%
Synovial Sarcoma (SS)	32	20.9%
Other	13	8.5%
**Tumor Subtype**		
**SS**	*n* = 32	
Monophasic	18	56.3%
Biphasic	5	15.6%
Missing Data	9	28.1%
**LPS**	*n* = 49	
Myxoid Round Cell Liposarcoma (MRCL)	31	63.3%
Well Differentiated/Dedifferentiated (WD/DD)	15	30.6%
Missing Data	3	6.1%
**LMS**	*n* = 34	
Uterine	3	8.8%
Non-Uterine	21	61.8%
Missing Data	10	29.4%
**Other**	*n* = 13	
Adenosarcoma	1	7.7%
Alveolar Rhabdomyosarcoma	1	7.7%
Carcinosarcoma	1	7.7%
Endometrial Stromal Sarcoma	1	7.7%
Epithelioid Sarcoma	1	7.7%
Ewing Sarcoma	1	7.7%
Fibromyxosarcoma	1	7.7%
Hemangioendothelioma	1	7.7%
Osteosarcoma	2	15.4%
Pleomorphic Rhabdomyosarcoma	2	15.4%
Spindle and Epithelioid Cell Sarcoma	1	7.7%
**Size of primary tumor (largest diameter**,** cm)**		
Mean (Range)	9.71 (1.2–40)	
Missing Data	40	

**Table 2:** Other demographics

Variable	*n* (total = 153)	%
Treatment		
Chemotherapy	39	25.4%
Radiotherapy	7	4.5%
Both	33	21.5%
Neither	72	47.0%
Missing Data	2	1.3%
**Grade**		
1	26	16.9%
2	55	35.9%
3	60	39.2%
Missing Data	12	7.8%
**Local recurrence**		
No	66	43.1%
Yes	46	30.0%
Missing Data	41	26.8%
**Developed metastasis**		
Yes	61	39.8%
No	53	34.6%
Missing Data	39	25.4%
**Alive at last date of follow up**		
Yes	80	52.3%
No	46	30.1%
Missing Data	27	17.6%

### B7-H3 expression in STS tumors

The expression of B7-H3 molecules was analyzed on 153 archived FFPE tumor samples. 97.3% of samples stained positive for B7-H3 (B7-H3 score > 0) and 69.2% of samples demonstrated high levels of expression (B7-H3 score ≥3) (Table 3). Overall survival appeared similar across both B7-H3 positivity and expression levels, although the analysis with respect to positivity was limited by the small number of patients with absent B7-H3 expression. (Appendices 4 and 5). All STS types appeared to have a high degree of positivity for B7-H3 expression. There was however variability in expression level by subtype, 34 patients with LMS were included, 33 (97%) had positive B7-H3 expression, with 21 (62%) having high expression and 13 (38%) having low or absent expression. All of the LPS patients had expression with 39 (80%) having high expression and only 10 patients having low expression. 24 of 25 patients with UPS had positive expression, with 20 patients (80%) having high expression levels. In patients with SS, 30 out of 32 had positive expression (94%) with 16 (50%) having positive expression. Thirteen patients had “other” STS tumors, 10 of which (77%) had high expression (Table 4). No significant differences in B7-H3 positivity or expression levels was found with prior treatment, tumor size, tumor grade or patient age. To look at the data in a different way, we grouped the samples in high versus low expressing groups. Using this classifiaction method B7-H3 expression levels differed significantly by sarcoma subtypes (*P* = 0.035, Appendices 2 and 3).

**Table 3:** B7-H3 positivity and expression

Variable	*n* (total = 153)	%
B7-H3 Positivity		
Negative (score = 0)	4	2.6%
Positive (score > 0)	149	97.3%
B7-H3 Expression		
Low (score < 3)	47	30.7%
High (score > = 3)	106	69.2%

**Table 4:** Summary of B7-H3 positivity/expression by tumor type

	LMS (*n* = 34)	LPS (*n* = 49)	UPS (*n* = 25)	SS (*n* = 32)	Other (*n* = 13)	*P*-Value
B7-H3 Positivity						0.333
Positive (score > 0)	33 (97%)	49 (100%)	24 (96%)	30 (94%)	13 (100%)	
Negative (score = 0)	1 (3%)	0 (0%)	1 (4%)	2 (6%)	0 (0%)	
B7-H3 Expression						0.035
High Expression (score ≥ 3)	21 (62%)	39 (80%)	20 (80%)	16 (50%)	10 (77%)	
Low Expression (score < 3)	13 (38%)	10 (20%)	5 (20%)	16 (50%)	3 (23%)	

*percentile of high/ low B7-H3 expression represent the ratio of high B7-H3 expression to total B7-H3 cases, P-values were calculated based on Fisher’s Exact Test

The Staining pattern of B7-H3 was evaluated in 153 tissue samples and both cytoplasmic and membranous expression were assessed (Appendix 12). Vessels surrounding the tumor samples were evaluated for B7-H3 expression in the tumor microenvironment (TME), and expression was found in 94.77% (145/153) of samples available.

Because B7-H3 is in the same family as PD-L1, we thought there might be a correlation of PD-L1 with high levels of B7-H3. PD-1 and PD-L1 data expresion data is included in Appendices 6 – 9. There was no correlation between either the positivity or level of B7-H3 expression with positivity or expression level of PD-L1 or PD-1 (Appendices 10 and 11).

## Discussion

Overexpression of B7-H3 has been previously identified in several STS subtypes [19, 23, 25, 34], although its implications on the pathogenesis and aggressiveness of disease is yet to be elucidated. In osteosarcoma, B7-H3 has been shown to affect the proliferation and metastasis of tumor cells. Some have observed that B7-H3 negatively correlated with the degree of tumor infiltration by CD8 + T lymphocytes, suggesting that it impairs T-cell-mediated immunity [24]. CAR-T cells directed at B7-H3 have shown effectiveness in eradicating osteosarcoma in preclinical studies [16, 34] and both monoclonal antibodies as well as antibody drug conjugates directed at B7-H3, are currently being investigated [18, 35, 36]. B7-H3 expression also occurs in Langerhans cell sarcoma, where it has been found to be coexpressed with tumor-associated PD-L1 [19]. 

The exact biological functions of B7-H3 have not yet been elucidated, and its implications in other cancer types highlight its dual costimulatory and coinhibitory abilities. Though overall survival appeared similar across both B7-H3 positivity and expression levels in our study, previous studies have shown that B7-H3 has important and often divergent prognostic significance in a number of malignancies. High B7-H3 expression in pancreatic cancer is associated with significantly prolonged postoperative survival [20]. In contrast, high B7-H3 expression in patients with gastric cancer correlates with a significantly lower 5 year survival rate and may serve as a useful biomarker of gastric tumor progression [21]. Likewise, B7-H3 expression by renal cell carcinoma or its TME was also significantly associated with increased mortality [22]. 

Results from our study suggest that B7-H3 is expressed across many STS subtypes; including LPS, SS, LMS, and UPS, and at high levels in the majority of cases. Expression of the molecule was recognized within both tumor tissue and the vasculature across subtypes, identifying it as an attractive target for therapy. However, any role it may play in the pathogenesis and immune evasion of sarcoma is unclear. This study did not show significant correlations of PD-L1/PD-1 coexpression positivity or expression level with B7-H3. As not a very high number of cases had negative or low expression of PD-1, PD-L1, or B7-H3, our study had limited power to assess the association between B7-H3 and PD-1/PD-L1. Larger sample size is warranted to fully evaluate the association between B7-H3 and PD-1/PD-L1.

This is the most comprehensive analysis of B7-H3 expression in sarcoma ever performed. However, our study had several important limitations. Median survival was calculated on the basis of outcomes from a historical database of patients with sarcoma and relied on adequate documentation in the electronic medical record. Our study population was subject to selection bias, as it contained many patients with more common sarcoma subtypes and lacked patients with rarer histologic types, such as chordoma or clear cell sarcoma, which could potentially underestimate the correlation between B7-H3 positivity/expression and sarcoma subtypes. It is intriguing to wonder whether B7-H3 might play a role in tumor pathogenesis and the TME in common sarcoma subtypes; however, this study looked only at expression and did not include a functional analysis. Even though the function and activity of B7-H3 remain unknown, high expression of B7-H3 in multiple sarcoma types makes it an attractive targetable cell surface molecule.

Furthermore, the association between B7-H3 expression and Progression Free Survival (PFS) and Disease-Free Survival (DFS) was not analyzed in this study. Nor did it assess differences in B7-H3 expression in certain patients with high-risk characteristics such as high grade, large tumor size, or patients presenting with deep to fascia tumors compared to the rest of the patient cohort, which would be interesting to assess in future studies. Lastly, the sample size of evaluated PD-1/PD-L1 cases may have underestimated a correlation between B7-H3 and PD-1/PD-L1.

Despite its limitations, these data suggest that B7-H3 is almost universally expressed in STS. Further, B7-H3 appears to be expressed at high levels in the majority of STS, supported by the inclusion of cases in this study representing the dominant subtypes of STS seen in practice. Even without a complete understanding of its role in the pathogenesis of STS, targeting B7-H3 could provide a viable general therapeutic strategy in STS management. The remarkable consistency of its expresion warrants further investiation of its role in STS and potential as a therapeutic target. Future studies assessing therapeutic efficacy of B7-H3 in STS are warranted.

## Electronic supplementary material

Below is the link to the electronic supplementary material.

Supplementary Material 1

## Data Availability

The datasets generated and/or analyzed during the current study are not publicly available but are available from the corresponding author on reasonable request.
