# Supplementary material for: B7-H3 is widely expressed in soft tissue sarcomas
Source: BMC Cancer. 2024 Oct 30;24:1336. doi: 10.1186/s12885-024-13061-4 (PMC11523878; doi:10.1186/s12885-024-13061-4)
Supplement: Supplementary file 1 — Supplementary Material 1 [file 12885_2024_13061_MOESM1_ESM.docx]

Appendix 1: "Other" Histologies

| **Other Soft Tissue Sarcomas** | **n=13** | **% out of total Other** | **% out of total cases included (N=153)** |
| --- | --- | --- | --- |
| Carcinosarcoma | 1 | 7.6% | 0.6% |
| Spindle & epithelioid cell sarcoma | 1 | 7.6% | 0.6% |
| Ewing Sarcoma | 1 | 7.6% | 0.6% |
| Alveolar Rhabdomyosarcoma | 1 | 7.6% | 0.6% |
| Pleomorphic Rhabdomyosarcoma | 2 | 15.3% | 1.2% |
| Endometrial Stromal Sarcoma | 1 | 7.6% | 0.6% |
| Hemangioendothelioma | 1 | 7.6% | 0.6% |
| Osteosarcoma | 2 | 15.3% | 1.2% |
| Epithelioid Sarcoma | 1 | 7.6% | 0.6% |
| Fibromyxosarcoma | 1 | 7.6% | 0.6% |
| Adenosarcoma | 1 | 7.6% | 0.6% |

Appendix 2: B7H3 positivity subgroup analysis

|  | **1: Negative (Score=0)** | **2: Positive (Score>0)** | **Total***** | **p value** |
| --- | --- | --- | --- | --- |
| **Age (Years) at Diagnosis** |  |  |  | 0.968^1^ |
| *N* | 3 | 113 | 116 |  |
| *N-Miss* | 1 | 36 | 37 |  |
| *Mean (SD)* | 47.6 (8.0) | 47.9 (14.7) | 47.9 (14.6) |  |
| *Range* | 42.3 - 56.7 | 14.1 - 86.3 | 14.1 - 86.3 |  |
| **Prior Treatment*** |  |  |  | 0.508^2^ |
| *Chemotherapy* | 0 (0%) | 39 (26%) | 39 (26%) |  |
| *Radiation* | 0 (0%) | 7 (5%) | 7 (5%) |  |
| *Both* | 0 (0%) | 33 (22%) | 33 (22%) |  |
| *Neither* | 3 (100%) | 69 (47%) | 72 (48%) |  |
| **Tumor Type*** |  |  |  | 0.333^2^ |
| *Leiomyosarcoma (LMS)* | 1 (25%) | 33 (22%) | 34 (22%) |  |
| *Liposarcoma (LPS)* | 0 (0%) | 49 (33%) | 49 (32%) |  |
| *Undifferentiated Pleomorphic*  *Sarcoma (UPS)* | 1 (25%) | 24 (16%) | 25 (16%) |  |
| *Synovial Sarcoma (SS)* | 2 (50%) | 30 (20%) | 32 (21%) |  |
| *Other* | 0 (0%) | 13 (9%) | 13 (8%) |  |
| **Grade*** |  |  |  | >0.99^2^ |
| *Grade 1* | 0 (0%) | 26 (19%) | 26 (18%) |  |
| *Grade 2* | 1 (33%) | 54 (39%) | 55 (39%) |  |
| *Grade 3* | 2 (67%) | 58 (42%) | 60 (43%) |  |
| **Size of Primary Tumor (Largest Diameter, cm)** |  |  |  | 0.438^1^ |
| *N* | 3 | 110 | 113 |  |
| *Mean (SD)* | 12.8 (7.3) | 9.6 (7.0) | 9.7 (7.0) |  |
| *Range* | 5.0 - 19.5 | 1.2 - 40.0 | 1.2 - 40.0 |  |

1. Linear Model ANOVA
2. Fisher’s Exact Test for Count Data

*Percentiles represent the proportion of the variables in the positive / negative B7-H3 expression categories.

**Missing variables’ data was removed from the table and reported in tables 1and 2.

***The Total does not include missing data for corresponding variables.

Appendix 3: B7H3 Expression subgroup analysis.

|  | **1: Low**** (Score<3)** | **2: High (Score>=3)** | **Total***** | **p value** |
| --- | --- | --- | --- | --- |
| **Age (Years) at Diagnosis** |  |  |  | 0.228^1^ |
| *N* | 34 | 82 | 116 |  |
| *Mean (SD)* | 45.4 (15.3) | 49.0 (14.2) | 47.9 (14.6) |  |
| *Range* | 14.1 - 70.3 | 18.2 - 86.3 | 14.1 - 86.3 |  |
| **Prior Treatment*** |  |  |  | 0.517^2^ |
| *Chemotherapy* | 14 (30%) | 25 (24%) | 39 (26%) |  |
| *Radiation* | 1 (2%) | 6 (6%) | 7 (5%) |  |
| *Both* | 12 (26%) | 21 (20%) | 33 (22%) |  |
| *Neither* | 19 (41%) | 53 (50%) | 72 (48%) |  |
| **Tumor Type*** |  |  |  | 0.035^2^ |
| *Leiomyosarcoma (LMS)* | 13 (28%) | 21 (20%) | 34 (22%) |  |
| *Liposarcoma (LPS)* | 10 (21%) | 39 (37%) | 49 (32%) |  |
| *Undifferentiated Pleomorphic Sarcoma (UPS)* | 5 (11%) | 20 (19%) | 25 (16%) |  |
| *Synovial Sarcoma (SS)* | 16 (34%) | 16 (15%) | 32 (21%) |  |
| *Other* | 3 (6%) | 10 (9%) | 13 (8%) |  |
| **Grade*** |  |  |  | 0.699^2^ |
| *Grade 1* | 9 (21%) | 17 (17%) | 26 (18%) |  |
| *Grade 2* | 18 (42%) | 37 (38%) | 55 (39%) |  |
| *Grade 3* | 16 (37%) | 44 (45%) | 60 (43%) |  |
| **Size of Primary Tumor (Largest Diameter, cm)** |  |  |  | 0.612^1^ |
| *N* | 33 | 80 | 113 |  |
| *Mean (SD)* | 9.2 (5.3) | 9.9 (7.6) | 9.7 (7.0) |  |
| *Range* | 1.5 - 22.0 | 1.2 - 40.0 | 1.2 - 40.0 |  |

1. Linear Model ANOVA
2. Fisher’s Exact Test for Count Data

*Percentiles represent the proportion of the variables in the low / high B7-H3 expression categories.

**Missing variables’ data was removed from the table and reported in tables 1and 2.

***The Total does not include missing data for corresponding variables.

****Low category of B7-H3 expression includes negative expression of B7-H3.

Appendix 4: B7H3 positivity and median survival


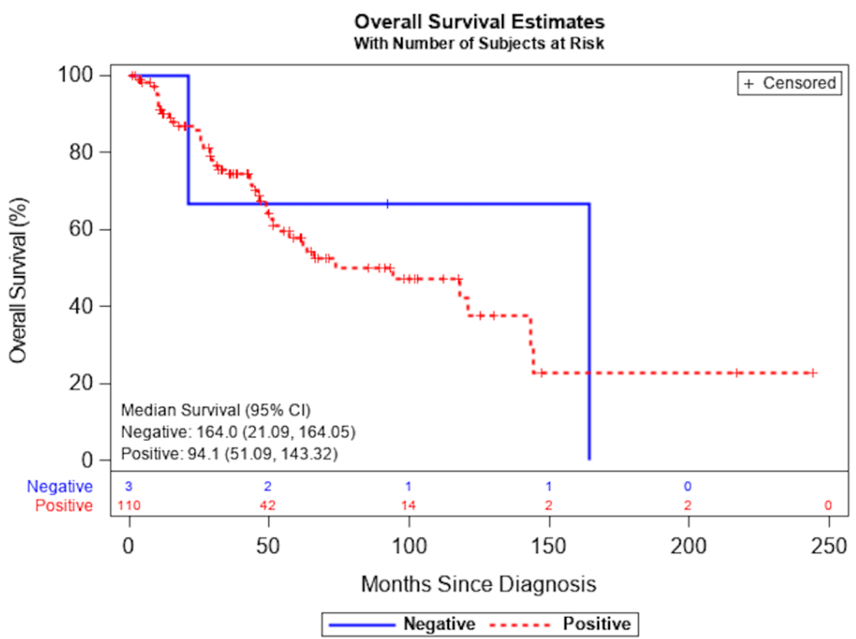


Appendix 5: B7H3 expression and median survival


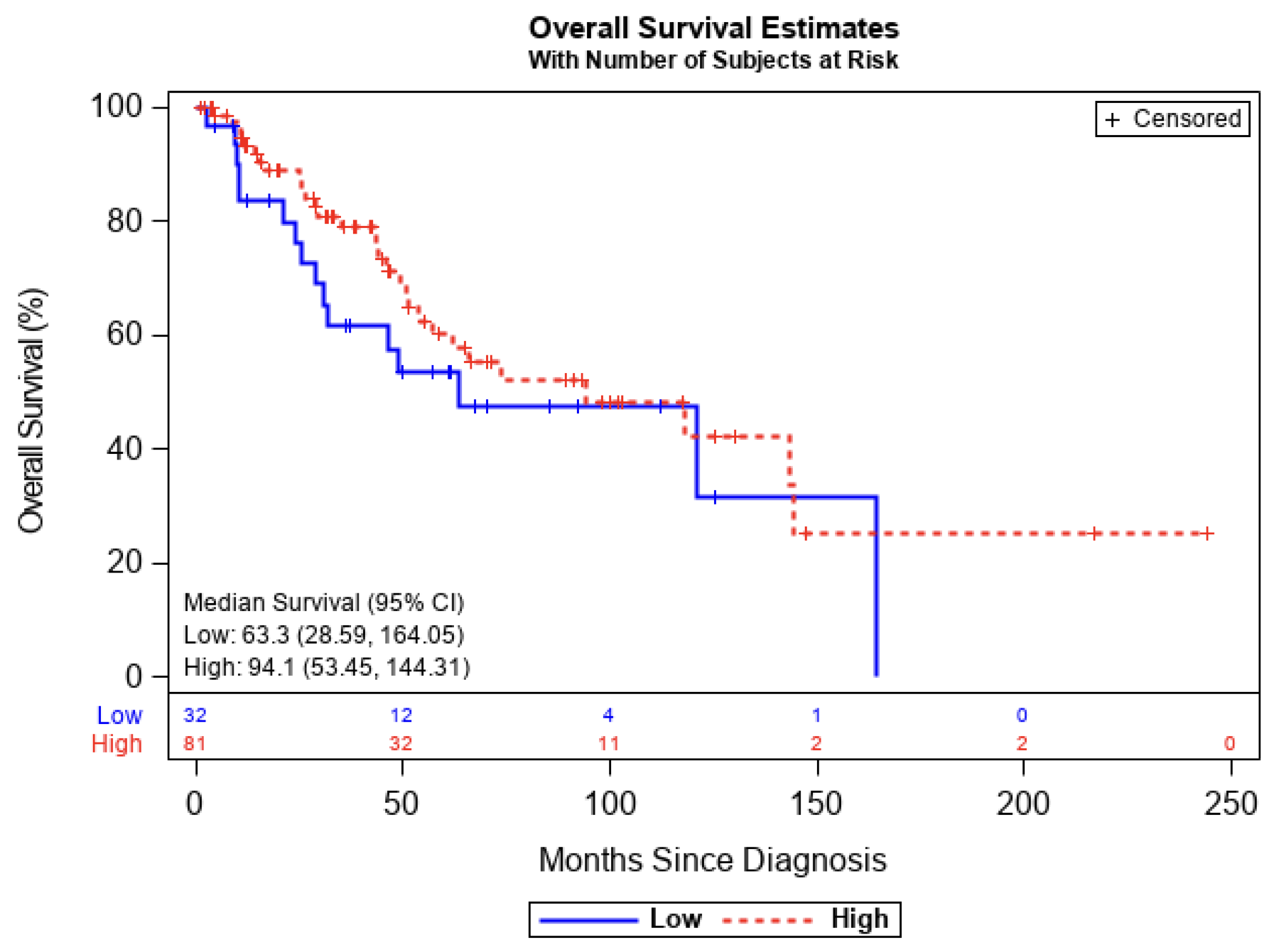


Appendix 6: PD-I positivity subgroup analysis

|  | **1: Negative (Score=0) (N=7)** | **2: Positive (Score>0) (N=72)** | **Total (N=79)**** | **p value** |
| --- | --- | --- | --- | --- |
| **Age (Years) at Diagnosis** |  |  |  | 0.622^1^ |
| *N* | 7 | 72 | 79 |  |
| *Mean (SD)* | 47.8 (9.8) | 50.6 (15.0) | 50.4 (14.6) |  |
| *Range* | 33.2 - 63.2 | 18.2 - 86.3 | 18.2 - 86.3 |  |
| **Prior Treatment** |  |  |  | 0.882^2^ |
| *Chemotherapy* | 2 (29%) | 18 (25%) | 20 (25%) |  |
| *Radiation* | 1 (14%) | 6 (8%) | 7 (9%) |  |
| *Both* | 1 (14%) | 15 (21%) | 16 (20%) |  |
| *Neither* | 3 (43%) | 33 (46%) | 36 (46%) |  |
| **Tumor Type** |  |  |  | 0.484^2^ |
| *Leiomyosarcoma (LMS)* | 3 (43%) | 16 (22%) | 19 (24%) |  |
| *Liposarcoma (LPS)* | 3 (43%) | 24 (33%) | 27 (34%) |  |
| *Undifferentiated Pleomorphic*  *Sarcoma (UPS)* | 1 (14%) | 18 (25%) | 19 (24%) |  |
| *Synovial Sarcoma (SS)* | 0 (0%) | 14 (19%) | 14 (18%) |  |
| **Grade** |  |  |  | 0.160^2^ |
| *Grade 1* | 3 (43%) | 12 (17%) | 15 (19%) |  |
| *Grade 2* | 1 (14%) | 29 (41%) | 30 (38%) |  |
| *Grade 3* | 3 (43%) | 30 (42%) | 33 (42%) |  |
| **Size of Primary Tumor (Largest Diameter, cm)** |  |  |  | 0.475^1^ |
| *N* | 7 | 72 | 79 |  |
| *Mean (SD)* | 8.0 (4.2) | 10.2 (7.8) | 10.0 (7.6) |  |
| *Range* | 2.6 - 15.0 | 1.2 - 40.0 | 1.2 - 40.0 |  |

1. Linear Model ANOVA
2. Fisher’s Exact Test for Count Data

*Missing variables’ data was removed from the table and censored during analysis. **The Total per variable does not include missing data for corresponding variables.

Appendix 7: PD-I expression subgroup analysis

|  | 1: Low** (Score<2) (N=38) | 2: High (Score>=2) (N=41) | Total***(N=79) | P value |
| --- | --- | --- | --- | --- |
| **Age (Years) at Diagnosis** |  |  |  | 0.049^1^ |
| *N* | 38 | 41 | 79 |  |
| *Mean (SD)* | 47.0 (12.2) | 53.5 (16.0) | 50.4 (14.6) |  |
| *Range* | 27.8 - 73.6 | 18.2 - 86.3 | 18.2 - 86.3 |  |
| **Prior Treatment** |  |  |  | 0.504^2^ |
| *Chemotherapy* | 7 (18%) | 13 (32%) | 20 (25%) |  |
| *Radiation* | 3 (8%) | 4 (10%) | 7 (9%) |  |
| *Both* | 8 (21%) | 8 (20%) | 16 (20%) |  |
| *Neither* | 20 (53%) | 16 (39%) | 36 (46%) |  |
| **Tumor Type** |  |  |  | 0.005^2^ |
| *Leiomyosarcoma (LMS)* | 9 (24%) | 10 (24%) | 19 (24%) |  |
| *Liposarcoma (LPS)* | 16 (42%) | 11 (27%) | 27 (34%) |  |
| *Undifferentiated Pleomorphic*  *Sarcoma (UPS)* | 3 (8%) | 16 (39%) | 19 (24%) |  |
| *Synovial Sarcoma (SS)* | 10 (26%) | 4 (10%) | 14 (18%) |  |
| **Grade** |  |  |  | 0.225^2^ |
| *Grade 1* | 10 (27%) | 5 (12%) | 15 (19%) |  |
| *Grade 2* | 14 (38%) | 16 (39%) | 30 (38%) |  |
| *Grade 3* | 13 (35%) | 20 (49%) | 33 (42%) |  |
| **Size of Primary Tumor (Largest Diameter, cm)** |  |  |  | 0.542^1^ |
| *N* | 38 | 41 | 79 |  |
| *Mean (SD)* | 10.5 (8.1) | 9.5 (7.2) | 10.0 (7.6) |  |
| *Range* | 1.2 - 39.2 | 2.0 - 40.0 | 1.2 - 40.0 |  |

1. Linear Model ANOVA
2. Fisher’s Exact Test for Count Data

*Missing variables’ data was removed from the table and censored during analysis.

**The Total does not include missing data for corresponding variables.

***Low category of PD-1 expression includes negative expression of PD-1.

Appendix 8: PD-LI positivity subgroup analysis

|  | **1: Negative (Score=0) (N=32)** | **2: Positive (Score>0) (N=47)** | **Total** (N=79)** | **p value** |
| --- | --- | --- | --- | --- |
| **Age (Years) at Diagnosis** |  |  |  | 0.007^1^ |
| *N* | 32 | 47 | 79 |  |
| *Mean (SD)* | 45.1 (12.8) | 53.9 (14.7) | 50.4 (14.6) |  |
| *Range* | 18.2 - 73.6 | 24.8 - 86.3 | 18.2 - 86.3 |  |
| **Prior Treatment** |  |  |  | 0.180^2^ |
| *Chemotherapy* | 9 (28%) | 11 (23%) | 20 (25%) |  |
| *Radiation* | 1 (3%) | 6 (13%) | 7 (9%) |  |
| *Both* | 4 (12%) | 12 (26%) | 16 (20%) |  |
| *Neither* | 18 (56%) | 18 (38%) | 36 (46%) |  |
| **Tumor Type** |  |  |  | 0.067^2^ |
| *Leiomyosarcoma (LMS)* | 8 (25%) | 11 (23%) | 19 (24%) |  |
| *Liposarcoma (LPS)* | 14 (44%) | 13 (28%) | 27 (34%) |  |
| *Undifferentiated Pleomorphic*  *Sarcoma (UPS)* | 3 (9%) | 16 (34%) | 19 (24%) |  |
| *Synovial Sarcoma (SS)* | 7 (22%) | 7 (15%) | 14 (18%) |  |
| **Grade** |  |  |  | 0.060^2^ |
| *Grade 1* | 10 (31%) | 5 (11%) | 15 (19%) |  |
| *Grade 2* | 12 (38%) | 18 (39%) | 30 (38%) |  |
| *Grade 3* | 10 (31%) | 23 (50%) | 33 (42%) |  |
| **Size of Primary Tumor (Largest Diameter, cm)** |  |  |  | 0.329^1^ |
| *N* | 32 | 47 | 79 |  |
| *Mean (SD)* | 9.0 (6.0) | 10.7 (8.5) | 10.0 (7.6) |  |
| *Range* | 1.2 - 29.0 | 2.0 - 40.0 | 1.2 - 40.0 |  |

1. Linear Model ANOVA
2. Fisher’s Exact Test for Count Data

*Missing variables’ data was removed from the table and censored during analysis. **The Total per variable does not include missing data for corresponding variables.

Appendix 9: PD-LI expression subgroup analysis

|  | **1: Low*** (Score<2) (N=52)** | **2: High (Score>=2) (N=27)** | **Total** (N=79)** | **p value** |
| --- | --- | --- | --- | --- |
| **Age (Years) at Diagnosis** |  |  |  | 0.041^1^ |
| *N* | 52 | 27 | 79 |  |
| *Mean (SD)* | 48.0 (13.7) | 55.0 (15.3) | 50.4 (14.6) |  |
| *Range* | 18.2 - 86.0 | 24.8 - 86.3 | 18.2 - 86.3 |  |
| **Prior Treatment** |  |  |  | 0.466^2^ |
| *Chemotherapy* | 15 (29%) | 5 (19%) | 20 (25%) |  |
| *Radiation* | 5 (10%) | 2 (7%) | 7 (9%) |  |
| *Both* | 8 (15%) | 8 (30%) | 16 (20%) |  |
| *Neither* | 24 (46%) | 12 (44%) | 36 (46%) |  |
| **Tumor Type** |  |  |  | < 0.001^2^ |
| *Leiomyosarcoma (LMS)* | 13 (25%) | 6 (22%) | 19 (24%) |  |
| *Liposarcoma (LPS)* | 23 (44%) | 4 (15%) | 27 (34%) |  |
| *Undifferentiated Pleomorphic*  *Sarcoma (UPS)* | 5 (10%) | 14 (52%) | 19 (24%) |  |
| *Synovial Sarcoma (SS)* | 11 (21%) | 3 (11%) | 14 (18%) |  |
| **Grade** |  |  |  | 0.018^2^ |
| *Grade 1* | 13 (25%) | 2 (7%) | 15 (19%) |  |
| *Grade 2* | 22 (43%) | 8 (30%) | 30 (38%) |  |
| *Grade 3* | 16 (31%) | 17 (63%) | 33 (42%) |  |
| **Size of Primary Tumor (Largest Diameter, cm)** |  |  |  | 0.048^1^ |
| *N* | 52 | 27 | 79 |  |
| *Mean (SD)* | 11.2 (8.5) | 7.6 (4.6) | 10.0 (7.6) |  |
| *Range* | 1.2 - 40.0 | 2.0 - 19.5 | 1.2 - 40.0 |  |

1. Linear Model ANOVA
2. Fisher’s Exact Test for Count Data

*Missing variables’ data was removed from the table and censored during analysis.

**The Total does not include missing data for corresponding variables.

***Low category of PD-1 expression includes negative expression of PD-1.

Appendix 10: Correlation between B7H3 and PD-1/PD-L1 positivity:

|  | **B7-H3 Negative (Score=0) (N=2)** | **B7-H3 Positive (Score>0) (N=77)** | **Total (N=79)*** | **p value** |
| --- | --- | --- | --- | --- |
| **PD-1 Positivity** |  |  |  | >0.99^1^ |
| *Negative (Score=0)* | 0 (0%) | 7 (9%) | 7 (9%) |  |
| *Positive (Score>0)* | 2 (100%) | 70 (91%) | 72 (91%) |  |
| **PD-L1 Positivity** |  |  |  | >0.99^1^ |
| *Negative (Score=0)* | 1 (50%) | 31 (40%) | 32 (41%) |  |
| *Positive (Score>0)* | 1 (50%) | 46 (60%) | 47 (59%) |  |

1. Fisher’s Exact Test for Count Data

*Missing variables’ data was removed from the table and censored during analysis.

Appendix 11: Correlation between B7H3 and PD-1/PD-L1 expression:

|  | **B7-H3 Low (Score<3) (N=16)** | **B7-H3 High (Score>=3) (N=63)** | **Total (N=79)*** | **p value** |
| --- | --- | --- | --- | --- |
| **PD-1 Expression** |  |  |  | >0.99^1^ |
| *Low (Score<2)* | 8 (50%) | 30 (48%) | 38 (48%) |  |
| *High (Score>=2)* | 8 (50%) | 33 (52%) | 41 (52%) |  |
| **PD-L1 Expression** |  |  |  | >0.99^1^ |
| *Low (Score<2)* | 11 (69%) | 41 (65%) | 52 (66%) |  |
| *High (Score>=2)* | 5 (31%) | 22 (35%) | 27 (34%) |  |

1. Fisher’s Exact Test for Count Data

*Missing variables’ data was removed from the table and censored during analysis.

Appendix 12: I B7H3 staining pattern

| 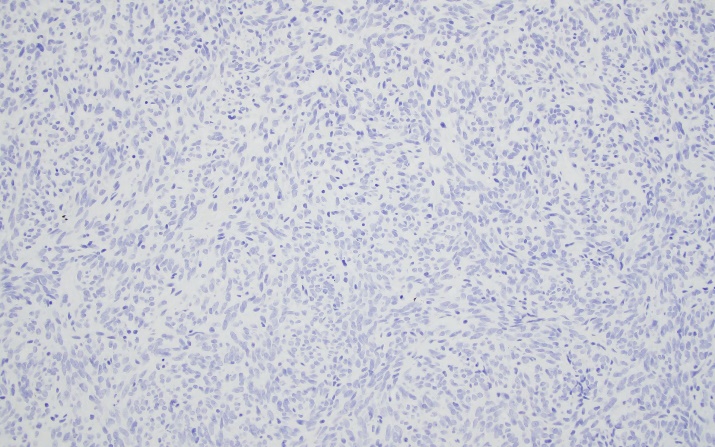 | 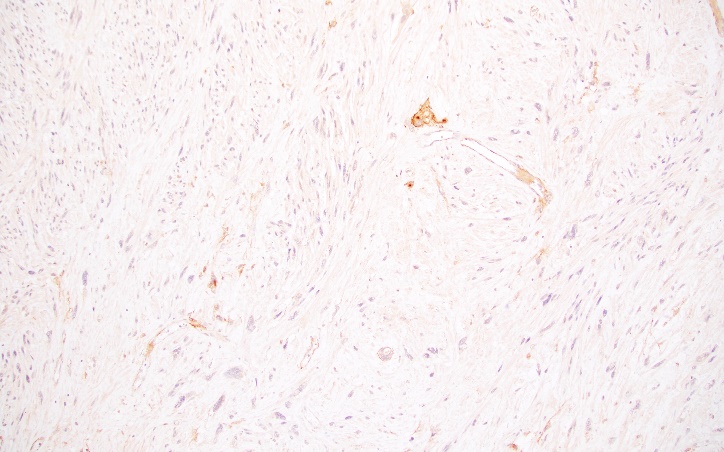 |
| --- | --- |
| 1. **Score 0** | 1. **Score 1** |
| 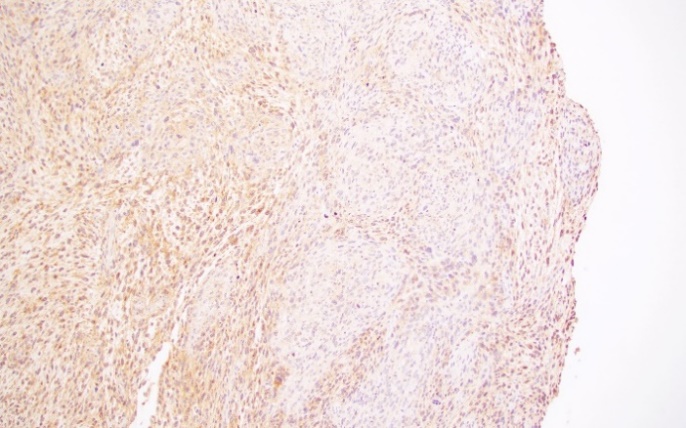 | 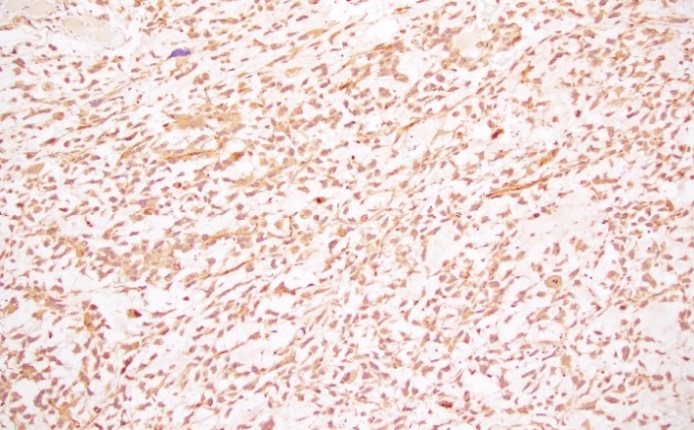 |
| 1. **Score 3** | 1. **Score 4** |
| Immunohistochemical staining for B7-H3 (A) Score 0 (0% positive cells): Negative staining for B7-H3 in the tumor cells. (B) Score 1: Positive membranous and cytoplasmic staining is seen in isolated tumor cells (between 0-25% of the tumor) as well as within normal endothelial cells. (C) Score 3: Patchy positive staining in approximately 50-7% of the tumor cells. (D) Score 4: Diffuse expression of B7-H3 with membranous and cytoplasmic positivity in the tumor cells as well as within the vascular endothelial cells. (between 75-100% of the tumor) (B7-H3 IHC, all images were captured at 10x magnification). | |
